# Decoding the Genetic and Structural Features of HPV16 E5 Oncogene in Cervical Cancer Isolates from Pakistan: A Pilot Study

**DOI:** 10.61186/ibj.3884

**Published:** 2023-08-23

**Authors:** Naureen Ehsan Ilahi, Nayyer Siddique, Muhammad Ibrahim Rashid, Mamoona Noreen, Sheeba Murad

**Affiliations:** 1 Atta-Ur-Rehman School of Applied Biosciences, National University of Science and Technology, H-12 Campus, Hucknall Road, Islamabad, Pakistan;; 2 Department of Biotechnology, Virtual University of Pakistan, Rawalpindi, Pakistan;; 3 Department of Molecular Biology and Genetics, Khyber Medical University, Peshawar, Pakistan;; 4 Department of Zoology, The Women University Multan, Multan, Pakistan

**Keywords:** Human papillomavirus 16, Oncogenes, Uterine cervical neoplasms

## Abstract

**Background:**

Many anogenital cancers are caused by high-risk HPV. The most common subtype is HPV16, which is prevalent in the world, including Pakistan. Various amino acid residues in HPV16 E5 are associated with high cell cycle progression and proliferation. Lack of studies on HPV16E5 in Pakistan prompted the current study. This is the first report on the occurrence of pathogenic E5 variant of HPV16 in tissue sections obtained from invasive cervical cancerous patients in Pakistan.

**Methods:**

A subset of 11 samples from HPV-positive biopsies were subjected to E5 gene amplification using PCR and analyzed using bioinformatics programs. The bioinformatics analysis detected mutations causing structural variations, which potentially contribute to the oncogenic properties of proteins.

**Results:**

The two-point mutations, C3979A and G4042A, observed in isolate 11 caused the substitution of isoleucine for leucine and valine at positions 44 and 65 in E5 protein. The rest of the isolates had Leu44Val65 amino acids. Intratypic variations and phylogenetic analysis revealed that the majority of the isolates were closely clustered with European-Asian lineage. Moreover, C3979A and G4042A contributed to higher degree of interactions with host receptors, i.e. EGFR.

**Conclusion:**

This is the first study reporting HPV16 variants in a Pakistani population based on variations in the E5 region. Our findings indicate that isolate 11 has a strong interaction with the intracellular domain of EGFR, which may enhance the generation of downstream signals. Since this was a pilot study to explore E5 gene mutation, future studies with large samples are absolutely needed.

## INTRODUCTION

High-risk types of HPVs play an important role in the etiology of cervical cancer ^[^^1^^-^^5^^]^. HPV16 is the predominant high-risk subtype responsible for the majority of HPV-caused malignancies. As a DNA virus, HPV16 genome is relatively stable, though it is believed to have adapted to its host. To date, three oncogenic proteins, E5, E6, and E7, have been identified. Genetic mutation in E5 gene may impact oncogenicity, antigenicity, and immunogenicity of the encoded proteins. Therefore, reports on the sequence analysis of HPV16 E5 from different parts of the world would be of importance.

Intratypic variants of HPV16 have formerly been categorized into four major lineages (A, B, C, and D) centered in E6, L1, L2, and LCR regions^[^^6^^,^^7^^]^ and 16 sub-lineages, including A-A4 (European-Asian), B1-B4 (African 1), C1-C4 (African 2), and D1-D4 (North American/Asian-American) lineages^[^^8^^]^. However, limited data on HPV16 lineages have been reported for Pakistan.

HPV16 E5 is a transmembrane and hydrophobic protein consisting of 83 amino acids^[^^9^^]^. E5 is known to associate with the membranes of Golgi apparatus and endoplasmic reticulum^[^^10^^]^ and participate in the early stages of the oncogenic process^[^^11^^-^^14^^]^. Recent studies have identified an important role for E5 in the later oncogenic stages. E5 has also been found to link with various host proteins such as MHC-I, Bap31, 16 kDa pore sub-unit of vacuolar-ATPase, and EGFR^[^^15^^-^^18^^]^. Previously, it has been shown that HPV16 E5 binds to EGFR^[^^18^^]^, and cells expressing 16 E5 increases mitogen-activated protein kinase activity, as well as improves responsiveness to EGF^[^^19^^,^^20^^].^ In recent years, there has been a growing interest in early E5 oncoprotein of HPV16 due to its sequence and functional diversity across phylogeny and especially due to its role as a viroporin; controlling vesicle trafficking, virion formation, ion homeostasis, and viral genome entrance 22357280^[^^21^^]^. E5 mRNA and protein have been found in low-grade cervical intraepithelial neoplasia, indicating that E5 actively participates in the early phases of neoplastic transformation^[^^11^^,^^12^^]^. In addition to E6 and E7, E5 is the third oncoprotein with the potential therapeutic target. E5 gene is divergent between different HPV types. Four different protein subgroups α to δ have been proposed for E5. HPV 16, 18, and 31, belong to the subgroups α^[^^22^^]^. The comparative small size of E5 makes it an attractive target for retrospective intratypic variant analysis^[^^23^^]^. Thus, intratypic amino acid variations may be relevant to the generation of speciﬁc immune responses, especially in the context of rational vaccine designing^[^^24^^]^. 

The current study was designed to identify sequence variation on E5 gene in HPV16-positive cervical carcinoma tissues and to determine whether specific genetic variations can aid in characterizing the HPV16 E5 variants present in our population.

## MATERIALS AND METHODS

**Sample collection and HPV genotyping**

This survey was conducted as an extension of our previously reported work^[^^25^^]^. A total of 49 paraffin-embedded biopsies were collected for this study. Among 37 tumors positive for HPV 16 and 18 DNA, 25 (51%) and 8 (16%) tumors were positive for HPV 16 DNA and HPV 18, respectively, and coinfection was detected in 4 (8%) tumors. Also, 12 tumors (24%) were negative for both HPV 16 and 18. Among the 37 positive samples, 11 samples were randomly selected to study E5 gene. Tissue samples were obtained from the Armed Forces Institute of Pathology, Rawalpindi (Pakistan) after the formal approval of the study. Histological types of epithelial cancers consisted of squamous cell carcinoma (n = 7) and nonkeratinizing squamous cell carcinoma (n = 4). DNA extraction from samples was performed manually by using the reported protocol^[25]^. Extracted DNA was screened for the presence of HPV by GP5^+^/GP6^+^ primers and HPV16 by new TS16 primer (Table 1) as described previously^[^^26^^]^.

**E5 gene amplification and sequencing**

E5 gene amplification was carried out by a primer set reported earlier by our group^[^^25^^]^ (Table 1). HPV16-positive samples were PCR amplified for E5 (252 bp) product, which was purified using the Gene JET PCR Purification Kit (Thermo Fisher Scientific, USA). The eluted and purified DNA samples of E5 gene were sent to Eurofins Genomics Europe (Germany) for sequence analysis. Sequencing was performed twice by both forward and reverse primers to ensure the nucleotide sequences.

**Table 1:** Primers used for HPV genotyping

**Primers**	**Sequence (5’-3)**	**Annealing** **temperature (**°C)	**Target gene**	**Product size (bp)**	**Reference**
GP5^+^/GP6^+^	F: TTTGTTACTGTGGTAGATAC	48	L1	150	^[^^27^^]^
	R: GAAAAATAAACTGTAAATCA				
New TS16	F: GGTCGGTGGACCGGTCGATG	58	HPV16 E6	96	^[^^28^^]^
	R: GCAATGTAGGTGTATCTCCA				
16-E5	F: GAATTCATGACAAATCTTGATACTG	54.6	HPV16 E5	252	^[^^25^^]^
	R: GGATCCTTATGTAATTAAAAAGCGT				

**Molecular variant detection in HPV16 E5 gene**

The obtained E5 sequences were aligned with each other using Multalin software to observe the nucleotide variation^[^^29^^]^. Sequences were also translated into protein using the translate tool of ExPASy server (http://web.expasy.org/translate/) and aligned to determine the amino acid alteration caused due to changes in the nucleotide. E5 sequences from Pakistani isolates (numbered 1-11) were then aligned with the representative sequences of different HPV16 lineages mentioned before^[^^8^^]^, i.e. lineage A: K02718.1^[^^30^^]^, AF536179.1^[^^31^^]^, HQ644236.1^[^^32^^]^, and AF534061.1^[^^31^^]^, lineage B (African 1 [AFR1]): AF536180.1^[^^31^^]^ and HQ644298.1^[^^32^^]^, lineage C (African 2 [AFR2]): AF472509.1^[31,33]^, and lineage D: AF402678.1^[^^33^^,^^34^^]^, AY686579.1^[^^33^^]^ and HQ644257.1^[^^32^^]^. Phylogenetic analysis was conducted using the freeware Phylogeny (http://www.phylogeny.fr/)^[^^35^^,^^36^^]^. 

**Bioinformatics analysis**

***Identification of epitopes***

After analyzing sequences and identifying mutations, we assessed the effects of these mutations on the structure of E5 protein. For this purpose, we first detected the epitopes of E5 protein and then distinguished both MHC I and II epitopes for the reference E5 sequence, isolates 10 and 11 using TepiTool (http://tools.iedb.org/ tepitool/). This tool predicted Class I and II MHC epitopes using IC_50_ value <500 nM.

**Structural analysis of the mutated E5 proteins**

Mutations at the DNA level can impact the protein structure by altering the amino acids, since some alterations are crucial in protein-protein interaction. To examine the effect of mutations on protein-protein interaction, we studied the structures of the most common isolates, i.e. 11 and 10, using the FALCON2 server (http://falcon.ictbda.cn:89/#home). To identify differences between these structures and the reference sequence, we made a comparison with the YASARA MUSTANG method using YASARA visualization software (version 23.9.29)^[^^37^^]^. To further investigate the impact of these mutated proteins on the EGFR downstream signaling pathway, we analyzed the interaction of these structures with the EGFR protein. The structures PDB id: 1M14 and 3NJP were obtained from the Protein Data Bank server (https://www.rcsb. org/)^[^^38^^]^. The interaction between the two structures was then analyzed using the docking web server ClusPro 2.0 (https://cluspro.bu.edu/login.php)^[^^39^^]^. The energy profiles were evaluated based on the electrostatic and van der Waals interactions using the following equation: E = 0.40Erep + -0.10Eatt + 600Eelec + 1.00EDARS. Models with lower energy were selected and visualized using Biovia Discovery Studio 2021 (https://www.3ds. com/products/biovia/discovery-studio/ligand-and-pharmacophore-based-design).

## RESULTS

**Fig. 1:**
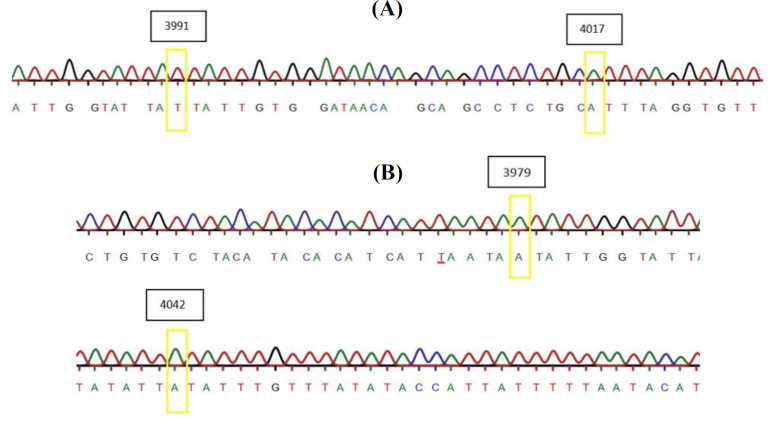
Sequencing chromatogram of mutations observed in (A) isolate 10 and (B) isolate 11

In the current study, HPV16 E5 gene was amplified from cervical cancer patients. Sequences obtained after PCR and DNA sequencing were deposited in the GenBank (accession nos. KX758540 and KX822075-84). Variations in nucleotide sequences of E5 open reading frame in the isolates are shown in Table 2 and Figure 1. Sequence variant from isolate 11 contained two point mutations, C3979A and G4042A, in comparison with the nucleotide sequence of HPV16 reference sequence (KF880690.1)^[^^40^^]^. In isolate 10, the following substitutions were observed: C3991T and G4017A. Both isolates 10 and isolate 11 belonged to the poorly and moderately differentiated squamous cell carcinoma.

**Fig. 2:**
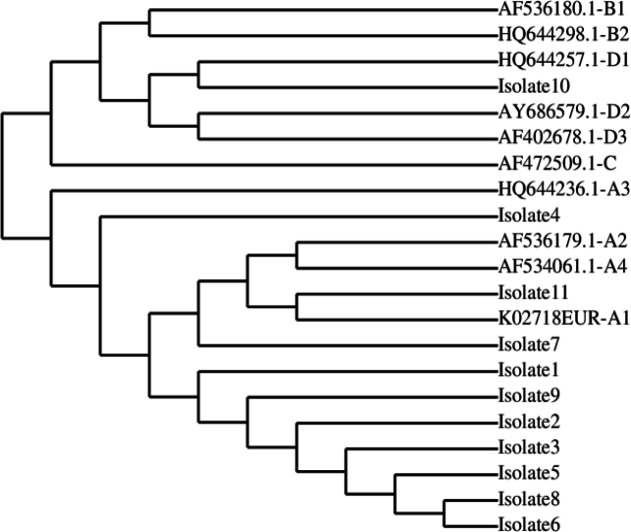
A phylogenetic tree based on HPV16 E5 analyses of 11 isolates (1-11) and reference sequences of different variants obtained from the GenBank database. A1-A4, B1-B2, C, and D1-D3 show European-Asian, African 1, African 2, and Asian American/North American lineages, respectively

A consensus sequence of the E5 was generated from all the isolates. Analysis of E5 nucleotide sequences showed 96-99% similarity between the study samples and the reference sequence (data not shown). Upon alignment with molecular variants of E5 originating from distinct lineages, the analysis unveiled the presence of five significant variations (Table 3). These variations exhibited a high degree of conservation within the majority of samples included in the current study, predominantly among lineage A (European-Asian lineage) variants. The majority of the African and American lineages had a different nucleotide at these positions (Table 3). On the basis of intratypic variations and phylogenetic analysis, it was observed that majority of the isolates were closely clustered with European-Asian lineage (Fig. 2). 

**Table 2:** Minor nucleotide variation observed in HPV16 E5 gene in the isolates and other HPV16 variant lineages

**Sequence no.**	**Isolate ** **no.**	**Nucleotide position in HPV16 E5 gene (3850-4101)**	**Type of variation and nucleotide change**	**HPV16 lineage with similar change**
1	11	3979	Transversion C A	A1
2	10	3991	Transition/transversion C/G T	B1, B2, D1
3	10	4017	Transition G A	D1-D3
4	11	4042	Transition G A	A1, A2

**Fig. 3:**
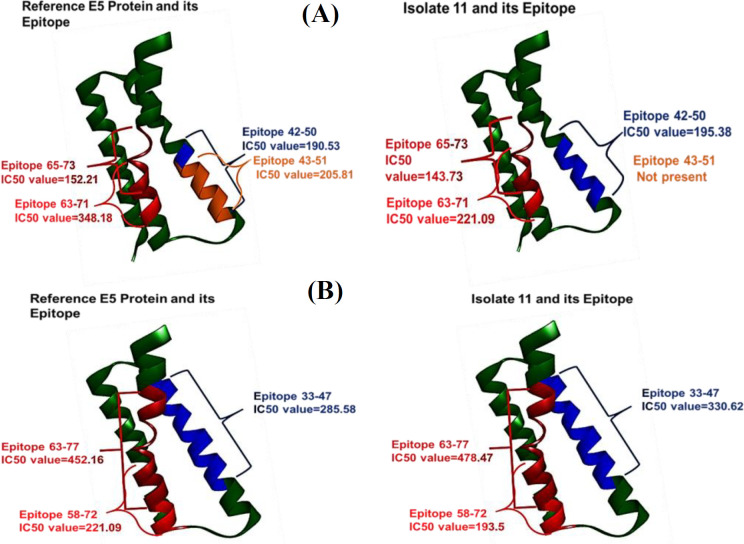
Different** (A) **MHC I and (B) MHC II epitopes of the reference E5 protein from isolate 11exhibiting notable variations in their IC_50_ values, which allow clear differentiation

The TepiTool software identified approximately 100 epitopes of the E5 proteins for each reference, as well as for the isolates 10 and 11 (Table 4 and Fig. 3). Epitopes containing mutations have different IC_50_ values compared to the reference sequence. Epitopes with an IC_50_ value higher than 250 nmol can evade the immune system, since a higher viral load is required to trigger an immune response.

**Fig. 4:**
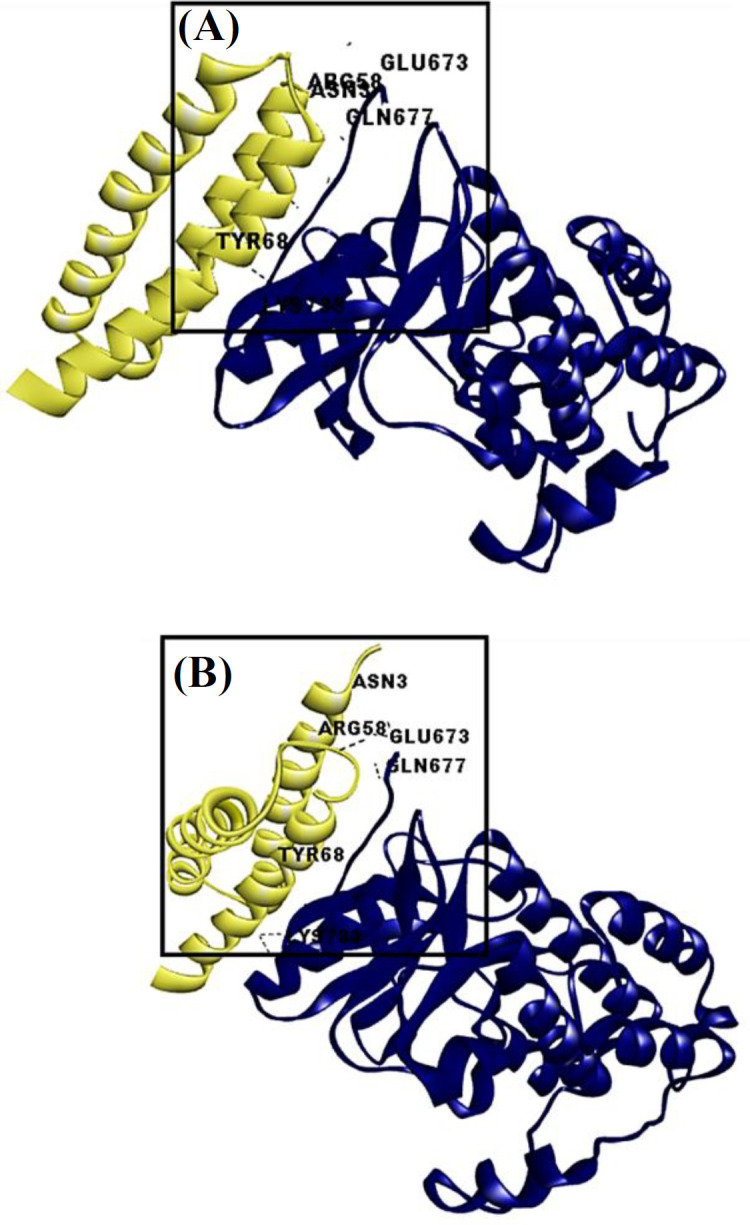
Superimposed structure of Wild type Reference E5 protein (green) and isolate 11 (yellow) shows a noticeable difference in their structures

**Fig. 5:**
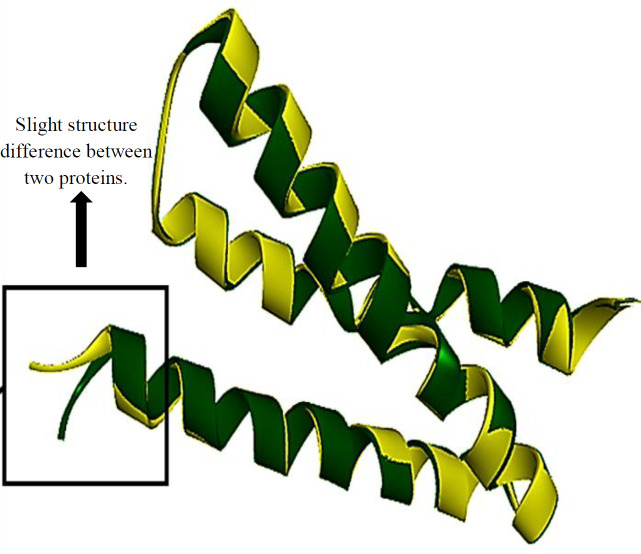
Interaction between the reference E5 protein (yellow) and EGFR (blue). (A) Reference E5 protein and EGFR and (B) isolate 11 and EGFR. The structural difference observed between the reference and isolate 11 may contribute to more interaction between the two proteins

To analyze the effects of mutations on the protein structures, a structure-structure alignment was performed. The findings revealed a difference with an RMSD value of 0.544 Å over 81 aligned residues and a 97.53% sequence identity (Fig. 4). This structural variation may contribute to the oncogenic properties of the different E5 variants. The interaction between E5 and the EGFR protein was examined through docking analysis. The results clearly demonstrated that the E5 protein of isolate 11 had more interactions (eight hydrogen bonds) with EGFR compared to the reference sequence (five hydrogen bonds). Moreover, the mutated isolate showed a stronger interaction with the intracellular domain of EGFR than the reference sequence, which may enhance the activation of the downstream signaling pathways (Fig. 5). 

## DISCUSSION

The current study aimed to investigate the presence of sequence variants of HPV16 E5 in cervical cancer biopsies of a Pakistani population. We confirmed the etiological involvement of HPV16 in the same cases of cervical cancer in our previous study using PCR and immunohistochemistry^[^^25^^]^. At present, we ampliﬁed the E5 gene by PCR from 11 HPV16-infected cervical cancer tissues. Heterogeneity in the viral oncogenes of HPV16 was found in a particular geographical region, which would be of functional significance. Studies have indicated that the prevalence of cervical cancer in different countries can be attributed to the distribution of specific viral variants of HPV genome, as HPV variants are differently dispersed among geographic regions^[^^41^^,^^42^^]^.

**Table 3:** Major nucleotide variation observed in HPV16 E5 gene in the isolates and other HPV16 variant lineages

**Sequence no.**	**Isolate no.**	**Nucleotide position in HPV16 E5 gene (3850-4101)**	**Type of variation and nucleotide change**	**HPV16 lineage with similar change**
1	1-10	3979	Transversion A C	All lineages except A1
2	1-9, 11	3991	Transition/transversion T/G C	A1, A2, A4
3	1-9, 11	4017	Transition A G	A1-A4, C, B1, B2
4	1-10	4042	Transition A G	A3, A4, C, D1-D3
5	1-11	4089	Transition C T	A1-A4

**Table 4:** Selected MHC I and MHC II epitopes covering the mutated region

**MHC I**	**41-49**	**42-50**	**43-51**	**61-69**	**63-71**	**64-72**	**65-73**
Reference E5	416.10	190.53	205.81	495.27	348.18	492.2	152.21
Isolate 10	416.10	190.53	205.81	495.27	348.18	492.2	
Isolate11	214.67	195.38		465.63	221.09	224.4	143.73
**MHC II**	**33-47**	**51-65**	**58-72**	**63-77**			
Reference E5	285.58	488.90	495.49	452.16			
Isolate 10	285.58	488.90	495.49	452.16			
Isolate11	330.62	472.44	193.50	478.47			

Molecular analysis of E5 gene in our samples revealed more conservation than variation in the nucleotide sequences. In a limited number of samples, only nucleotide substitutions with no deletion/insertion were identified. Four-point mutations were also observed in the isolates 10 (C3991T and G4017A) and 11 (C3979A and G4042A). In isolate 11, there were two non-synonymous mutations, which led to the replacement of leucine (L) at position 44 and valine at 65 by isoleucine (I) amino acid. Formerly, a functional analysis of this mutation revealed the importance of leucine and valine as α-helix stabilizers at the mentioned positions; however, substitution at amino acid 44 and 65 by isoleucine, a helix destabilizer, affected the structural integrity of E5 protein^[^^43^^]^. All other amino acids were fully conserved in the isolates. Similar to all E5 proteins of A9 group of papillomaviruses, the consensus sequences from the isolates belonged to E5α family^[^^22^^]^. 

In a previous study, similar to our study, no substitutions have been noted within amino acids 11-24^[^^44^^]^. This region forms a stretch of hydrophobic residues, potentially representing a transmembrane helical region. This transmembrane region has been introduced as the most conserved segment of E5 among HPV types of the A9 HPV group^[^^45^^]^. The sequence conservation observed in E5 can be attributed to the fact that E5 sequence variants have more than 98% similarity among their nucleotide sequences^[^^46^^]^. Additionally, the fact that almost identical strains have been found in unrelated people living in geographically different places without any known interaction is evidence of the sequence conservation in HPV16 E5 variations. Our study showed that HPV16 E5 variants in Pakistan are mostly related to one distinct group of HPV16 variants (European-Asian type). Sequence variations in E5 have not formerly been used to characterize the variants. Therefore, we attempted to connect the variations in E5 of different lineages to the ones included in our study. Interestingly, a high prevalence of European lineage found in the isolates, indicating a possible epidemiological link between Europe and Pakistan regarding the dissemination of HPV16 infections in Pakistan. Nevertheless, due to the small sample size, this finding should be verified by larger studies.

Our research focused on bioinformatics analysis of sequences, with particular emphasis on identification of epitopes for the reference sequence and the two primary isolates (10 and 11), due to their notable mutations. The epitope selection was based on the targeting the ones with significant mutations, guided by the understanding that E5 of HPV16 physically interacts with the heavy chain component of MHC-I antigen. This interaction retains HLA -I in Golgi bodies and endoplasmic reticulum prevents its transport to the cell surface, which consequently interfers with the immune presentation of viral peptides and reduces recognition by cytotoxic T lymphocytes^[^^47^^,^^48^^]^.

Our study assessed the structural variations resulting from the mutations C3979A and G4042A, specifically examining the interaction between E5 proteins and human EGFR. E5 protein activates EGFR, which triggers downstream signaling pathways, a phenomenon previously linked to protection against apoptosis^[^^49^^]^. Altogether, our findings suggest the potential activation of downstream signaling pathway due to a strong interaction of mutant E5 with EGFR. This dual focus on epitope identification and structural variations enhances our understanding of how HPV16 E5 may modulate immune recognition and downstream signaling and offers valuable insights for future research and potential therapeutic interventions. 

## DECLARATIONS

### Acknowledgments

We are obliged to the Higher Education Commission of Pakistan for providing a chance to conduct research. 

### Ethical statement

This article does not contain any studies with human participants or animals performed by any of the authors.

### Data availability

The raw data supporting the conclusions of this article are available from the corresponding author upon reasonable request. 

### Author contributions

NEI: conceptualization, experimentation, formal analysis, original draft, writing-review, and editing; NS and MI: bioinformatics analysis, writing-review, and editing; MN: formal analysis and experimentation; SM: funding acquisition, project administration, supervision, validation, and resources.

### Conflict of interest

None declared.

### Funding/support

A recurring budget of Attar-Ur-Rahman School of Applied Biosciences was provided by National University of Sciences and Technology (Islamabad, Pakistan) for the financial support of this project.
